# Use of online resources by undergraduate medical students at College of Medicine, Majmaah University, Kingdom of Saudi Arabia

**DOI:** 10.1371/journal.pone.0255635

**Published:** 2021-08-04

**Authors:** Khalid M. Alabdulwahhab, Syed Yousaf Kazmi, Waqas Sami, Khaled Nasser Almujel, Mohammed Hamed Alanazi, Khalid Falah Alanazi, Abdullah Meshal Moyana, Mohammad Shakil Ahmad, Tariq A. Alasbali, Fahd Al Alwadani

**Affiliations:** 1 Department of Ophthalmology, College of Medicine, Majmaah University, Al-Majmaah University, Al-Majmaah, Kingdom of Saudi Arabia; 2 Department of Pathology, College of Medicine, Majmaah University, Al-Majmaah University, Al-Majmaah, Kingdom of Saudi Arabia; 3 Department of Community Medicine & Public Health, College of Medicine, Majmaah University, Al-Majmaah University, Al-Majmaah, Kingdom of Saudi Arabia; 4 Medical Student, College of Medicine, Majmaah University, Al-Majmaah University, Al-Majmaah, Kingdom of Saudi Arabia; 5 Department of Ophthalmology, College of Medicine, Al-Imam Mohammad Ibn Saud Islamic University, Riyadh, Kingdom of Saudi Arabia; 6 Department of Ophthalmology, College of Medicine, King Faisal University, Hofuf, Kingdom of Saudi Arabia; King Saud University, SAUDI ARABIA

## Abstract

The current pandemic has revolutionized medical education with a rapid shift to online teaching and learning strategies. The students have coped by turning to the online resources to keep pace with the change. To determine the type and practice of online resources used by undergraduate medical students and compare the use of online resources with gender and GPA. This was a cross-sectional study in which an online self-administered questionnaire was used to evaluate the type and practices of the online resources used by the medical students during the Covid-19 pandemic. Complete enumeration sampling method was used to collect the data from 180 medical students studying at College of Medicine, Majmaah University, Saudi Arabia. One hundred and thirty students (72.2%) were unaware of the free online resources offered by the University. Most students (58.3%, n = 105) consulted peers for online references. Male students preferred PowerPoint presentations and consulting online resources for studying as compared to the females, whereas females preferred to study textbooks predominantly as compared to males (p = 0.005). Male students significantly shifted to the online resources during the COVID-19 pandemic as compared to females (p = 0.028). Students with the highest GPA scores shifted to online educational resources during pandemic. A significant proportion of the undergraduate medical students at College of Medicine, Majmaah University used online educational resources for learning. We recommend that the college administration for deliberation with the medical educationalists for necessary curricular amendments and taking necessary steps to make the college Academic supervision and mentorship program more proactive to meet the challenges of students’ use of online educational resources.

## Introduction

The turn of the millennium has seen some colossal advances in information technology worldwide. The introduction of the latest technologies, including laptops, smartphones, tabs, and fast internet speed, has revolutionized the lives of individuals worldwide. Medical education has also changed in the past decade to keep pace with the changing technologies and availability of superior software programs and apps that offer tutors and students a new horizon in understanding complex medical topics. The use of technology in education had already been in practice in developed countries around a decade ago, yet its acceptance by the educators was lagging behind the learners [1]. The current Covid-19 pandemic has accelerated the acceptance of technology usage in medical education to a new level [2]. Not only the teaching and learning but the assessment has been shifted online, a concept unimaginable some ten to fifteen years back.

The introduction of social media like Facebook, Twitter, Instagram, etc., has brought the world in the grasp of a small smartphone. The students now have access to the best graduates from the finest medical institutes in the world. Apart from social media pedestals, focused medicine-related academic networks also came to the horizon in the past decade. These include ResearchGate, Academia.edu, eMedicine, Medscape, and other programs that also targeted medical literature like Google scholars, Linked In, etc. [3]. The introduction of Wikipedia and YouTube as ancillary aid to medical resources has shifted medical learning to a new level. Studies conducted in recent times have shown that the information contained in these online resources are not always accurate and authentic [4, 5]. Many international studies in the western world in recent years were conducted to probe into the knowledge of the type of online resources used by the medical students [6–8].

The Kingdom of Saudi Arabia (KSA) has also kept pace with the world in this digital transformation and, in fact, is one of the leading countries to embrace such changes. Few studies conducted in recent times in KSA also points towards the changing perspective about students’ usage of online resources compared to conventional hard copy textbooks [9, 10]. Due to the dearth of national studies on this topic, we decided to carry out a study with the objectives to determine the various types of online resources utilized by the medical students in the college of medicine Majmaah University during Covid-19 pandemic and to observe any association between the gender and GPA scores to the type of online resources usage by the medical students.

## Materials and methods

A cross-sectional study was conducted at the College of Medicine Majmaah University from 1^st^ January to 31^st^ March 2021. The study participants included undergraduate medical students of both genders enrolled with the college of medicine. As per the latest E-Register statistics of the Students’ Affair section, the total number of enrolled students in the college of medicine, Majmaah University, were 210 (146 males and 64 females) at the time of the study, and all were included. There was no exclusion criterion for the participants. The medical program at college of medicine Majmaah University starts from second year and concludes at sixth year of training. The first year, also called the preparatory year, is under different patronage of Deanship of Common First Year. Therefore, only the medical students from Medical Program of second year to sixth year were included in the study. During study period of January to March 2021, the students in pre-clinical phase were attending block modules of Basic Therapeutics, Movement & its Control, Endocrine and Reproduction, Clinical skills foundation and two longitudinal modules belonging to Complementary and alternative medicine and Research. On the other hand, the students in Clinical phase were participating in Family Medicine, Dermatology, Psychiatry, Emergency Medicine, and Surgery blocks. The complete enumeration sampling technique was used for data collection. In this regard, an online self-administered google form was shared with the study participants to collect the response. The purpose of the study, confidentiality of the participants, and their free will to participate was explained in the introductory part of the questionnaire. A self-prepared questionnaire was used that was formulated by extensively reviewing the literature. The content validity was checked by three experts in the field who marked each question based on relevance and representativeness, after making the changes as advised by the experts, the final questionnaire was disseminated to the students. The first part of the questionnaire consisted of General information like age, year of studies, gender, and GPA score of the respondents. The GPA score of the students at the Majmaah University is adjusted by the students’ affairs section at the conclusion of each semester. Because our study finished in March 2021, approximately two months before the conclusion of second semester, therefore, we included the adjusted GPA scores of first semester for our study, i.e., September to December 2020.

The second part of the questionnaire comprised mostly closed-ended questions with either yes or no response, e.g., Did you shift to online resources during the COVID-19 pandemic? Yes/ No. Some questions were open-ended, e.g., Which online e-resource do you frequently use from Majmaah University? While some questions were semi-close-ended, e.g., From whom do you get the information about the use of online resources for medical studies? Participants were required to answer either Peers/ Teachers or mention others. The Internal Consistency Reliability (ICR) of the questionnaire was measured using Cronbach Alpha, the value of 0.71 shows that the questionnaire had a good reliability.

The ethical approval was obtained from Majmaah Research Institutional Ethics Committee of Basic & Health Science Research Center Majmaah (MUREC-Jan.21/COM-2021/19-5). Participation was purely voluntary, and the participants were explained the purpose of the study. The informed consent was obtained electronically. All data were kept confidential and utilized only for this study.

The data was entered and analyzed using IBM SPSS Statistics 26. Qualitative variables (age, gender, use of online resources etc.) were reported as frequencies and percentages. Pearson Chi-square and Fisher Exact tests were applied to observe associations between sociodemographic and GPA data. In case of a significant Chi-square test, post-Hoc test was applied to compare which group percentage differed using the Bonferroni adjustment. A p-value of <0.05 was considered statistically significant.

## Results

Out of 180 students who responded to the survey (response rate = 85.71%), female students comprised 68 (37.8%) while males were 112 (62%). Most of the students, 124 (68.9%), belonged to the age group of 21–23 years, followed by 40 (22.2%) who belonged to the age group of 18–20 years, while 16 (8.9%) fell in the age group of 24–26 years. Regarding GPA, most of the students 88 (48.9%) had it between 4.01–5.0, 42 (23.3%) had a GPA between 3.51–4.0, slightly more than one-fifth had it between 3.01–3.5, and 19 (10.6%) had a GPA of less than 3. Results are presented in Table 1.

**Table 1:** Demographic and educational characteristics of the students.

Characteristics	n (%)
**Age**	
18–20	40 (22.2)
21–23	124 (68.9)
24–26	16 (8.9)
**Gender**	
Female	68 (37.8)
Male	112 (62.2)
**GPA**	
2.5–3.0	19 (10.6)
3.01–3.50	31 (17.2)
3.51–4.0	42 (23.3)
4.01–5.0	88 (48.9)

Table 2 depicts the practices of different study resources as identified by the medical students. The majority of them (53.3%, n = 96) preferred to consult PowerPoint presentations of the lectures prepared by the tutors, while 53 (29.4%) searched online learning resources for their studies, whereas consulting traditional textbooks was the least preferred method (17.2%) only. Most of the students used Tablets 114 (63.3%) for searching the online resources, followed by laptops 47 (26.1%), while only 19 (10.6%) used smartphones for this purpose. During the COVID-19 pandemic, the bulk of the students (64.4%, n = 116) shifted to online resources for their academic development. As stated by the students, the primary aim of searching online references was to gain knowledge 104 (57.8%) while 63 (35%) used them to pass the examination.

**Table 2:** Practices of study resources usage by the students.

The practices of study resources usage by the students	n (%)
**Normally which type of resources do you consult more frequently for your medical studies?**	
Lecture PPT	96 (53.3)
Online	53 (29.4)
Traditional textbooks	31 (17.2)
**Which device do you use for consulting online resources?**	
Laptops	47 (26.1)
Smartphones	19 (10.6)
Tablets	114 (63.3)
**Did you shift to online resources during the COVID-19 pandemic?**	
No	64 (35.6)
Yes	116 (64.4)
**What is the main aim of using online resources?**	
To gain knowledge	104 (57.8)
To pass the exam	63 (35.0)
Both	5 (2.8)
Others	8 (4.4)

**Fig 1:**
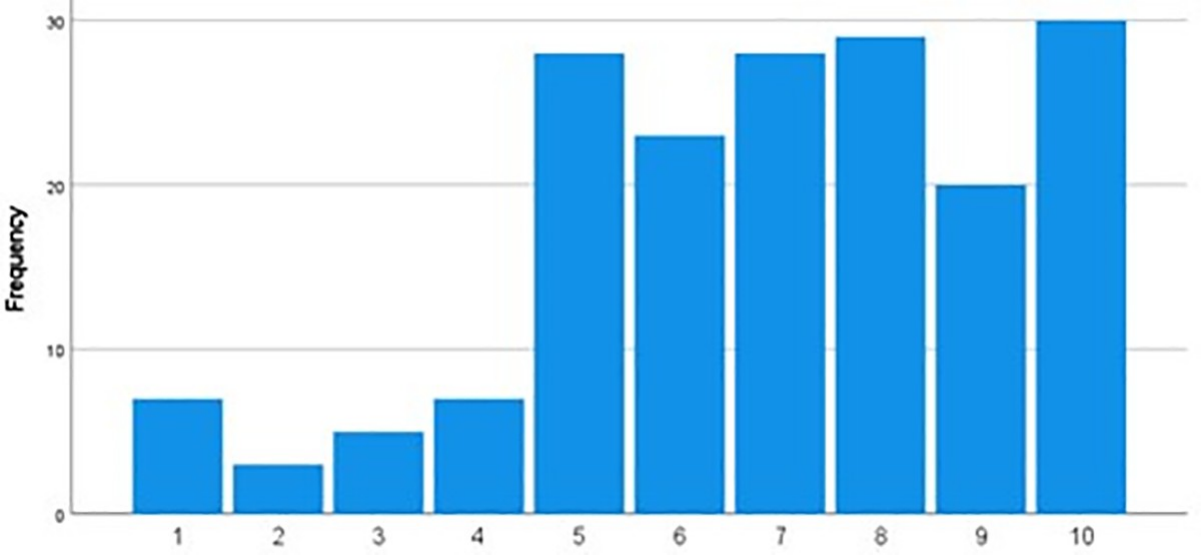
Likert scale response of the medical students’ experience of using online resources.

Table 3 highlights the awareness and attitude of the students towards the use of different online resources for their academic growth. The majority of the respondents (73.3%, n = 132) believed that the online resources were more useful than the traditional textbooks. A vast majority of students (85.6%, n = 154) used online resources because these were more user-friendly than the traditional textbooks. In response to the question, “Do you visit the university central library for resources?”, 118 (65.6%) replied in negative. Most of the students (72.2%, n = 130) were unaware of the electronic resources offered by the Majmaah University for the students. Responding to the question, “Do you use free online Q bank resources for your study?” 109 (60.6%) replied “No” while 71 (39.4%) replied in affirmative, of whom those who used Q bank most of them 27 (38.1%) consulted PassMedicine. Around 75 (41.7%) students had subscribed to the online lecture courses, while 86 (47.8%) of the respondents were using medical apps for online learning. When the students were asked, “From whom do you get the information about the use of online resources for medical studies?” majority of them 105 (58.3%) replied peer groups, whereas 65 (36.1%) got the information from the teachers, and a small percentage 5.6% obtained this information from the social media. In addition, Google was the most frequently used free search engine by the students, followed by YouTube, PubMed, Medscape, Mayo Clinic, Medscape, Wikipedia, ResearchGate, and others. Fig 1 depicts the Likert scale response of the medical students’ experience of using online resources. Results showed that most of the students (72.7%, n = 131) expressed satisfaction with using the online resources during the COVID-19 pandemic period.

**Table 3:** Awareness and attitude of the students towards the use of different online resources for their academic growth.

Questions	n (%)	Questions	n (%)
**Do you find online resources more useful than traditional textbooks?**		**Do you use free online Q bank resources for your study?**	
No	48 (26.7)	No	109 (60.6)
Yes	132 (73.3)	Yes	71 (39.4)
		**If yes, then which of the following free Q banks do you use?**	
		ExamDoctor	19 (26.7)
		PassMedicine	27 (38.1)
		PassTest	25 (35.2)
**Do you find online resources more user-friendly than traditional textbooks?**		**From whom do you get the information about the use of online resources for medical studies?**	
No	26 (14.4)	Teachers	65 (36.1)
Yes	154 (85.6)	Peers	105 (58.3)
		Social Media	10 (5.60)
		**Do you visit the University’s central library for resources?**	
		No	118 (65.6)
		Yes	62 (34.4)
**Are you aware of online e-resources offered by Majmaah university?**		**Which of the following free online resources/search engines do you like to visit most frequently?**	
No	130 (72.2)	Google	134 (74.4)
Yes	50 (27.8)	YouTube	39 (21.7)
		PubMed	23 (12.8)
**Do you use medical apps for online**		Medscape	22 (12.2)
**reference?**		Mayo Clinic	19 (10.5)
No	94 (52.2)	Wikipedia	10 (5.5)
Yes	86 (47.8)	ResearchGate	6 (3.3)
		Osmosis	2 (1.1)
**Do you have a subscription to online**		Ambos	1 (0.5)
**lecture courses?**		MdWeb	1 (0.5)
No	105 (58.3)		
Yes	75 (41.7)		

Results presented in Table 4 showed that predominantly male students preferred PowerPoint presentations and online resources for learning as compared to the females (post-hoc Bonferroni adjusted p-value <0.001). In contrast, females preferred to study textbooks predominantly as compared to males (p = 0.005). Similarly, male students significantly shifted to the online resources during the COVID-19 pandemic compared to females (p = 0.028). However, no significant association was observed between gender and device used for consulting online resources (p = 0.144), finding online resources more useful than traditional textbooks (p = 0.091), finding online resources more user-friendly than traditional textbooks (p = 0.719), visiting the university central library for resources (p = 0.433) and being aware of online e-resources offered by Majmaah University (p = 0.286).

**Table 4:** Association between gender and online learning resources questions.

Question	Response	Female	Male	Total	p-value
		n (%)	n (%)		
**Normally which type of resources do you consult more frequently for your medical studies?**	Lecture PPT	26 (27.1)	70 (72.9)	96	0.005*
	Online	25 (47.1)	28 (52.9)	53	
	Traditional textbooks	17(54.8)	14 (45.2)	31	
**Which device do you use for consulting online resources?**	Laptop	22 (46.8)	25 (53.1)	47	0.144
	Smartphone	4 (21.0)	15 (80.0)	19	
	Tabs	42 (36.8)	72 (63.2)	114	
**Did you shift to online resources during the COVID-19 pandemic?**	No	31 (48.4)	33 (51.6)	64	0.028*
	Yes	37 (31.8)	79 (68.2)	116	
**Do you find online resources more useful than traditional textbooks?**	No	23 (47.9)	25 (52.1)	48	0.091
	Yes	45 (34.1)	87 (65.9)	132	
**Do you find online resources more user-friendly than traditional textbooks?**	No	9 (34.6)	17 (65.4)	26	0.719
	Yes	59 (38.3)	95 (61.4)	154	
**Do you visit the University’s central library for resources?**	No	47 (39.8)	71 (60.2)	118	0.433
	Yes	21 (33.8)	41 (66.2)	62	
**Are you aware of online e-resources offered by Majmaah university?**	No	46 (35.3)	84 (64.7)	130	0.286
	Yes	22 (44.0)	28 (56.0)	50	

*Statistically significant at 5% level of significance.

Results presented in Table 5 showed that students with GPAs between 4.1–5.0 significantly shifted to the online resources during the COVID-19 pandemic compared to the students having other GPA values (Bonferroni adjusted p = 0.001). However, GPA was not significantly associated with the type of resources students consulted more frequently for their medical studies (p = 0.332), device used for consulting online resources (p = 0.528), finding online resources more useful than traditional textbooks (p = 0.481), finding online resources more user-friendly than traditional textbooks (p = 0.495), visiting the university central library for resources (p = 0.143) and being aware of the online e-resources offered by Majmaah University (p = 0.358).

**Table 5:** Association between GPA and online learning resources questions.

Question	Response	GPA				Total	p-value
		2.5–3.0	3.1–3.5	3.51–4.0	4.1–5.0		
		n (%)	n (%)	n (%)	n (%)		
**Normally which type of resources do you consult more frequently for your medical studies?**	Lecture PPT	10 (10.4)	16 (16.7)	28 (29.2)	42 (43.8)	96	0.332
	Online	7 (13.2)	7 (13.2)	9 (17.0)	30 (56.6)	53	
	Traditional textbooks	2 (6.5)	8 (25.8)	5 (16.1)	16 (51.6)	31	
**Which device do you use for consulting online resources?**	Laptop	5 (10.6)	5 (10.6)	11 (23.4)	26 (55.3)	47	0.528
	Smartphone	1 (5.3)	6 (31.6)	3 (15.8)	9 (47.4)	19	
	Tablet	13 (11.4)	20 (17.5)	28 (24.6)	53 (46.5)	114	
**Did you shift to online resources during the COVID-19 pandemic?**	No	14 (21.9)	13 (20.3)	14 (21.9)	23 (35.9)	64	0.001*
	Yes	5 (4.3)	18 (15.5)	28 (24.1)	65 (56.0)	116	
**Do you find online resources more useful than traditional textbooks?**	No	6 (12.5)	7 (14.6)	8 (16.7)	27 (56.3)	48	0.481
	Yes	13 (9.8)	24 (18.2)	34 (25.8)	61(46.2)	132	
**Do you find online resources more user-friendly than traditional textbooks?**	No	3 (11.5)	5 (19.2)	3 (11.5)	15 (57.7)	26	0.495
	Yes	16 (10.4)	26 (16.9)	39 (25.3)	73 (47.4)	154	
**Do you visit the University’s central library for resources?**	No	8 (6.8)	20 (16.9)	29 (24.6)	61 (51.7)	118	0.143
	Yes	11 (17.7)	11 (17.7)	13 (21)	27 (43.5)	62	
**Are you aware of online e-resources offered by Majmaah university?**	No	15 (11.5)	22 (16.9)	34 (26.2)	59 (45.4)	130	0.358
	Yes	4 (8.0)	9 (18.0)	8 (8.0)	29 (58.0)	50	

*Statistically significant at 5% level of significance.

## Discussion

The current COVID-19 pandemic has seen a swift transition in medical education from conventional face-to-face teaching, learning, and assessment to a dramatic online shift [11]. This has resulted in ease for the students and the faculty members alike in convening the sessions that could be easily implemented online. Depending upon the nature of the module, the learning and teaching methodology in College of Medicine Majmaah University was shifted online wholly or partially since the onset of the Covid-19 pandemic. Therefore, the students spent more time on the devices for learning activities which is speculated to prompt them to utilize handy online resources during Covid-19 pandemic times. Our planned sample size was 210, which comprised the whole number of students in the College of Medicine, Majmaah University, KSA. However, only 180 students filled the online questionnaire, representing around 86% of the expected value. This turnout was due to the early termination of the second semester on the direction of the Ministry of Education, and subsequently, earlier rescheduling of the final examinations. The predominance of male students 68.9% (n = 112) in our study was expected as this reflected the ratio between male and female students at the College of Medicine, Majmaah University (70% to 30%).

Our results showed that many students (53.3%, n = 96) preferred PowerPoint presentations for referencing while 29.4% (n = 53) consulted online content for resources. Similar results were observed in a study conducted in 2017 at the King Abdul Aziz University, Jeddah, by Jameel et al. on a sample size of 347, wherein most students preferred lecture handouts/ PowerPoint presentations [9]. Predominately male students in our study relied on lectures PowerPoint presentation along with online resources, while female students primarily utilized traditional textbooks for their learning. A study conducted in 2016 at King Saud University, Riyadh, with a sample size of 176 medical students, it was found that female students utilized online resources for their learning more than the male counterparts, while the preferences for lectures, PowerPoints, and traditional textbooks as a learning resource by both genders were almost similar [10]. The likely explanation for this contrast could be because this study was conducted before the Covid-19 pandemic when the online resources were not as popular and attractive for the students as they are now. In our study, the consultation of the traditional textbooks was the least preferred mean of learning by the medical students (17.2%). This conforms with various research and studies conducted in the past where students preferred online open educational resources more than the traditional textbooks [12, 13]. The main reasons for lack of preference for traditional textbooks could be the cost, weight, difficulty in finding a topic and cross-referencing, availability, etc.

Around 64.4% (n = 116) of our undergraduates shifted to online resources for their studies during the pandemic. Many of our students reside in Riyadh, and they frequently make a trip from Riyadh to Majmaah to attend classes that take around two hours for one trip. Therefore, due to the transition of online teaching, students had more time at home, which encouraged them to consult the online resources. It is also a fact that the newer online educational resources targeting different specialties have surfaced during the Covid-19 pandemic, which is another cause for the shift of our students to consult these resources [14]. The students appreciated that they found online educational resources more useful (73.3%, n = 132) and user-friendly (85.6%, n = 154) than the traditional textbooks. These findings are similar to a study conducted in 2017 in Sydney, Australia, on a sample size of 98 medical students in a clinical block, where more than 95% of the students found the local and other online resources helpful and convenient compared to the traditional textbooks [15].

Only a minority of the students (34.4%, n = 62) visited the University’s central library, which held a plethora of traditional textbooks and a cache of the electronic database. In 2018, the College of Medicine Majmaah University was shifted from the old building that stationed the Central library to the latest campus, which, although close, is still few kilometers away. This might be a reason for the lack of the students’ visits to the University Central Library. Our results showed that a significant majority of students (72.2%, n = 130) were unaware of any e-resources offered by the Majmaah University. These results were unanticipated with such high rates as this finding contrast to a study conducted in 2015–16 on 300 students of the medical and dental college from Al-Jouf University, Skaka, KSA, in which 75.5% of the medical students were aware of and utilizing the electronic resources offered by the college administration [16]. Saudi Digital Library was introduced in 2010, and it accommodates more than 240,000 full-text eBooks and access to millions of journal articles worldwide [17]. The main reason for this lack of awareness might be attributed to the students’ acquisition of information about online educational resources mainly from the peers (58.3%, n = 105), whereas only a fraction of them gets this information from their teachers (36.1%, n = 65). This dependency upon the peer to get advice about learning online resources is a universal phenomenon and noted in other international studies also. For example, a study conducted in 2017 at the University of Melbourne, Parkville, Australia, showed that the students sought advice for learning resources from peers more frequently than from the teachers (Mean response 3.9 vs. 2.8 on a 5-point Likert scale) [18]. As we can see that 39.4% (n = 71) of our study participants were using online QBanks for their studies, which included PassMedicine (38.1%, n = 27), PasTest (35.2%, n = 25), and ExamDoctor (26.7%, n = 19). Literature shows that these QBanks are very popular among the medical students for their preparation for USMLE examination [19], which means that our students consult quality online QBanks for their exam’s preparation; a fact reflected by the College of Medicine Majmaah University’s ranking among the top three universities of KSA in her graduates passing percentage of the SMLE.

Google and YouTube were the most frequently utilized search engine/ online educational resources by our students (74.4%, n = 134 and 21.7%, n = 39 respectively). This finding is consistent with national and international studies worldwide, where Google and YouTube were important tools for extracting the requisite information by the medical students [20, 21]. Our students also utilized online free medical platforms like Medscape, Mayo Clinics, ResearchGate, MdWeb, which are considered authentic sites for online referencing. Also, a good number of the medical undergraduates (41.7%, n = 75) had a subscription to paid online courses, which is an encouraging sign because spending money for learning reflects commitment and professionalism.

Overall, the majority of the respondents from our College of Medicine were satisfied with the online educational resources for learning (Mean of 10-point Likert Scale Response 6.9). In our study, apart from the consultation of traditional textbooks and lecture handouts, we did not find any statistically significant variations among both genders about the awareness, attitude, and practice of the use of learning resources. When we compared the students’ responses across different GPA ranges, we found that during the Covid-19 pandemic, the students with the highest GPA grades between 4.1–5 significantly shifted to consult the online educational resources than the students having lower GPA. This finding is significant because it reflected the attitude of the high-performing students who consider online resources more important to secure better grades in the examination. A study conducted on a large sample size of 21,822 students at the University of Georgia, the USA, in 2017 concluded that online open educational resources significantly improve Grades achievement by the students at the end of the course [22].

### Recommendations

We recommend that the course coordinators and faculty members start incorporating the authentic online educational resources in the study guides to help the students scroll the reliable and focused information. We also advocate the inculcation of new themes in the curriculum related to authentic online educational resources. This will boost the medical students’ confidence in smart and efficient browsing of electronic online resources. We also suggest that the modules like Medical Learning Skills, Medical Informatics, Ethics and Professionalism should include special visits to the University Central Library to motivate students to utilize available print and digital resources. Similarly, frequent demonstrations to the students should be convened by library staff on Saudi Digital Library. The college Academic supervision and mentorship program should strive hard in maintaining efficient liaison with the students to guide online educational resources.

### Limitations of the study

The main limitation of our study was being cross-sectional in nature, and there is a possibility of a reporting bias. As this study is conducted in one institution only, therefore, the results might not be generalized to other institutions, however, these results are of keen interest for higher authorities in Majmaah University. Future study, preferably having a comparative design and comprising a larger sample size involving multiple institutes should be undertaken to ascertain the students’ true practices of using online learning resources.

## Conclusions

Keeping pace with the rapid transformation in Medical Education in the time of Covid-19 pandemic, a considerable majority of students shifted to online educational resources for learning purposes with a significant preponderance of higher GPA scores and male gender. Google and YouTube remained the primary sources for searching online learning resources. Despite majority of the students’ satisfaction was with the online educational resources in our study, a bulk of students were neither aware of the free online e-resources offered by Majmaah University nor visited the central library. This trend reflects the students’ reliance primarily on their peers for advice. Necessary changes in the curriculum should be incorporated to address the new challenges of the learning and teaching resources, while tutors should guide and motivate the students about using authentic online educational resources.

## Supporting information

S1 Questionnaire (DOCX)

S1 Datasheet (SAV)
