# Supplementary material for: Use of online resources by undergraduate medical students at College of Medicine, Majmaah University, Kingdom of Saudi Arabia
Source: PLoS One. 2021 Aug 4;16(8):e0255635. doi: 10.1371/journal.pone.0255635 (PMC8336793; doi:10.1371/journal.pone.0255635)
Supplement: S1 Questionnaire — (DOCX) [file pone.0255635.s001.docx]

ONLINE RESOURCES USAGE BY MEDICAL

UNDERGRADUATE STUDENTS

General Information

# Age Years, Gender, Male ☐ Female ☐ Year of Study, 2^nd^ ☐ 3^rd^ ☐ 4^th^ ☐ 5^th^ ☐ 6^th^ ☐ GPA Score

Normally which type of resources do you consult more frequently for your medical studies?

Traditional textbooks ☐ Lecture PPT ☐ Online ☐

Which device do you use for consulting online resources?

Laptop ☐ Smartphones ☐ Tabs ☐

Did you shift to online resources during the COVID-19 pandemic?

Yes ☐ No ☐

If Yes, then do you experience improvement in your overall performance?

Yes ☐ No ☐

What is the main aim of using online resources?

To pass the exam ☐ To gain knowledge ☐ Other

Do you find online resources more useful than traditional textbooks?

Yes ☐ No ☐

Do you find online resources more user-friendly than traditional textbooks?

Yes ☐ No ☐

Do you visit the university central library for resources?

Yes ☐ No ☐

Are you aware of online e-resources offered by Majmaah university?

# Yes ☐ No ☐

If Yes, then which online e-resource do you frequently use from Majmaah University?

Which of the following free online resources/ search engines do you like to visit most frequently?

# Google ☐ Wikipedia ☐ YouTube ☐ PubMed ☐ Medscape ☐ MayoClinic ☐ Research Gate ☐ Others (Please specify)

On a scale of 1 to 10, how do you evaluate your experience with online studying during the Covid-19 pandemic? (1 signifies extremely unsatisfied while 10 means extremely satisfied)

Do you use free online Q bank resources for your study?

Yes ☐ No ☐

If Yes, then which of the following free Q banks do you use?

PassMedicine ☐ PasTest ☐ OnExamination ☐ ExamDoctor ☐ NEJM Knowledge + ☐ Others (Please Specify)

Do you have a subscription to online lecture courses?

Yes ☐ No ☐

Do you use medical apps for online reference?

# Yes ☐ No ☐

If Yes, which app do you use?

From whom do you get the information about the use of online resources for medical studies?

# Teachers ☐ Peer groups ☐ Others

Can

you

name

some

major

obstacles

to

use

online

resources?
